# Orientation-Controlled Electrocatalytic Efficiency of an Adsorbed Oxygen-Tolerant Hydrogenase

**DOI:** 10.1371/journal.pone.0143101

**Published:** 2015-11-18

**Authors:** Nina Heidary, Tillmann Utesch, Maximilian Zerball, Marius Horch, Diego Millo, Johannes Fritsch, Oliver Lenz, Regine von Klitzing, Peter Hildebrandt, Anna Fischer, Maria Andrea Mroginski, Ingo Zebger

**Affiliations:** 1 Institut für Chemie, Technische Universität Berlin, Straße des 17. Juni 135 & 124, D-10623, Berlin, Germany; 2 Biomolecular Spectroscopy/LaserLaB Amsterdam, Vrije Universiteit Amsterdam, De Boelelaan 1083, NL-1081 HV Amsterdam, The Netherlands; 3 Institut für Biologie/Mikrobiologie, Humboldt-Universität zu Berlin, Chausseestraße 117, D-10115, Berlin, Germany; 4 Institut für Anorganische und Analytische Chemie, Albert-Ludwigs-Universität Freiburg, Albertstrasse 21, D-79104, Freiburg, Germany; Martin-Luther University Halle-Wittenberg, GERMANY

## Abstract

Protein immobilization on electrodes is a key concept in exploiting enzymatic processes for bioelectronic devices. For optimum performance, an in-depth understanding of the enzyme-surface interactions is required. Here, we introduce an integral approach of experimental and theoretical methods that provides detailed insights into the adsorption of an oxygen-tolerant [NiFe] hydrogenase on a biocompatible gold electrode. Using atomic force microscopy, ellipsometry, surface-enhanced IR spectroscopy, and protein film voltammetry, we explore enzyme coverage, integrity, and activity, thereby probing both structure and catalytic H_2_ conversion of the enzyme. Electrocatalytic efficiencies can be correlated with the mode of protein adsorption on the electrode as estimated theoretically by molecular dynamics simulations. Our results reveal that pre-activation at low potentials results in increased current densities, which can be rationalized in terms of a potential-induced re-orientation of the immobilized enzyme.

## Introduction

**Fig 1:**
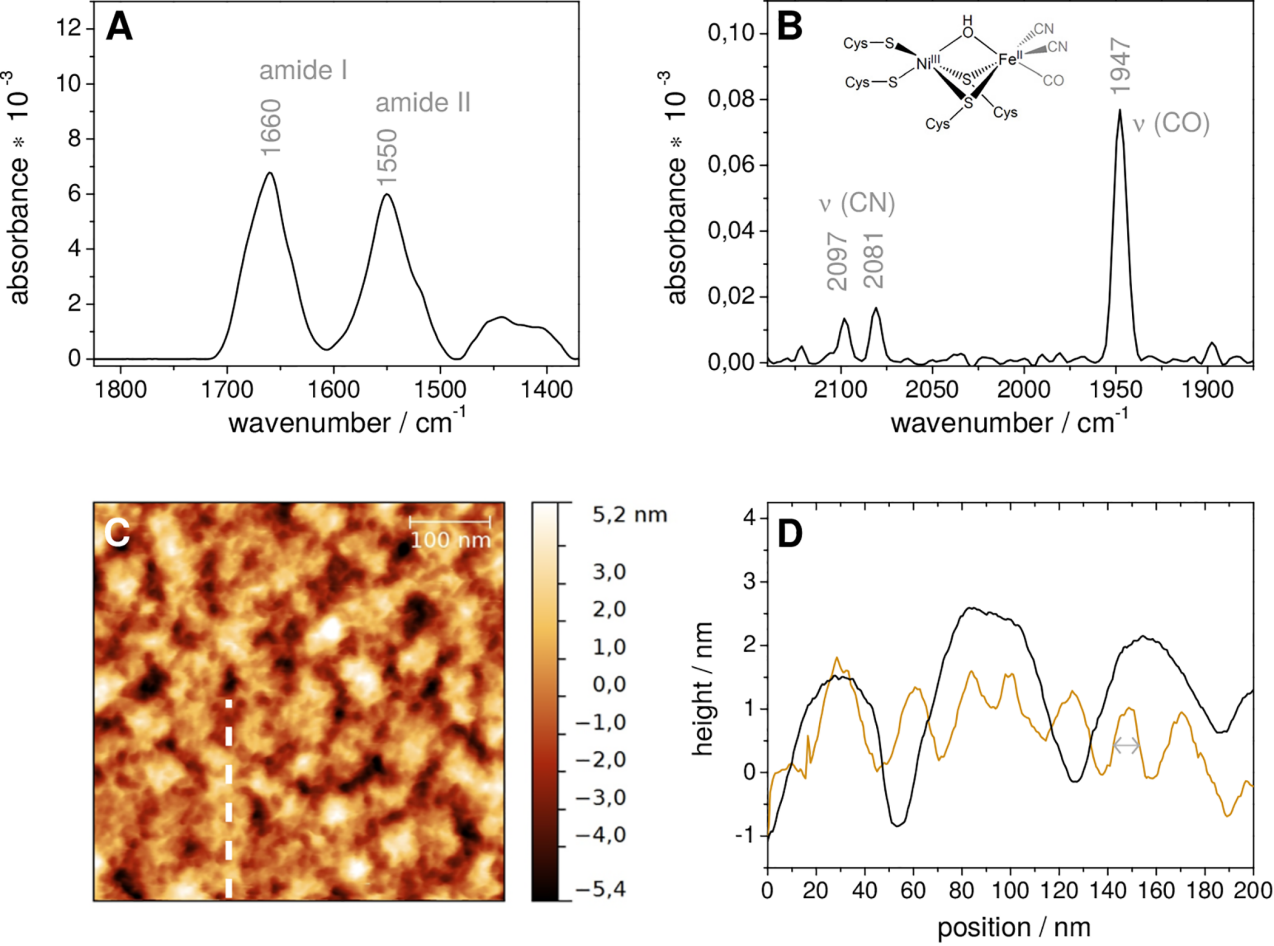
Top: SEIRA spectra of the *Strep*-tagged *Re* MBH, immobilized on a nanostructured Au surface coated with a self-assembled monolayer (SAM) of 6-amino-1-hexanethiol. Spectra are shown for (A), the amide mode region and, (B), the CO and CN stretching region of the active site. A structural depiction of the active site in the oxidized Ni_r_-B state is shown in the inset. Bottom: (C) Non-contact-mode AFM topographic mapping of the SAM-modified Au surface after completed MBH immobilization. The dashed vertical line indicates the course of the height profiles shown in (D) for immobilized *Re* MBH (orange line) and prior to immobilization (black line; see Fig B in S1 Appendix). The grey double-headed arrow indicates the space of a single MBH molecule.

[NiFe]-hydrogenases catalyze the reversible cleavage of molecular hydrogen into electrons and protons. In view of the increasing importance of H_2_-based technologies in energy storage and conversion, the biotechnological potential of these enzymes is extensively explored. In this context, oxygen-tolerant hydrogenases capable of H_2_-cycling in the presence of oxygen are particularly interesting. Prominent examples are the membrane-bound [NiFe]-hydrogenases (MBHs) from *Ralstonia eutropha* (*Re*) H16, *Aquifex aeolicus* (*Aa*), and *E*. *coli* [1–4]. The *Re* MBH is a heterodimeric protein, consisting of a large subunit (HoxG), harboring the [NiFe] active site, and a small subunit (HoxK), which contains the corresponding electron transfer chain. This electron relay consists of one [4Fe4S], one [3Fe4S], and an unusual [4Fe3S] cluster, the latter of which is crucial for the oxygen tolerance of the enzyme [5–7]. The Ni and Fe ions of the bimetallic catalytic center are coordinated by four cysteine residues. In addition, one CO and two CN^−^ ligands bind to the Fe (Fig 1B) [7,8].

In order to exploit the full potential of oxygen-tolerant hydrogenases for technological application, e.g., in bio-fuel cells, the key challenge is to accomplish appropriate enzyme immobilization that preserves the native structure and function of the enzyme and ensures efficient electrical coupling between the active site and the electrode. Since the early studies of direct enzyme coupling on pyrolytic graphite electrodes, [9] researchers have made substantial progress in designing biocompatible surfaces to study different hydrogenases, ranging from self-assembled monolayers (SAMs) to protein-tethered lipid bilayer [10] or polymeric scaffolds [11]. In the present work we chose SAMs because they provide an ideal platform to apply our integrated approach. The electrical wiring of the enzyme to the electrode is then probed by protein film voltammetry (PFV) [9].

However, PFV *per se* does not provide direct structural information regarding the involved catalytic species or direct insights into the mode of surface adsorption. Consequently, other methods have to be applied to gain insights into these aspects, which are essential for the design and optimization of hydrogenase-based bioelectronic devices. Recently, a systematic study using atomic force microscopy (AFM), PFV, and polarization modulation-infrared reflection absorption spectroscopy (PM-IRRAS) revealed that the H_2_-driven catalytic currents of the oxygen-tolerant *Aa* MBH correlate with the electrode surface functionalities used for enzyme immobilization on gold. The latter also influence interactions of the protein with the SAM, which were suggested to be governed by the C-terminal hydrophobic tail of the *Aa* MBH [12]. More recently, extended molecular dynamics (MD) simulations for this enzyme revealed a weak and fluctuating dipole moment [13]. These studies, however, were carried out in bulk solution, thereby neglecting the influences of enzyme-surface interactions (*vide supra*). Electrochemical control measurements performed in the same study were accomplished by using positively and negatively charged modified graphite electrodes [13], thereby also excluding direct comparison with the previous immobilization study of the enzyme [12]. While the PM-IRRAS technique applied in this former study provided information about the orientation of the adsorbed protein by examining the amide I and II bands of the protein backbone, the active site could not be probed. In this respect, surface enhanced infrared absorption (SEIRA) spectroscopy represents a powerful alternative. Besides monitoring enzyme adsorption and orientation, this technique can also probe the stretching modes of the active site diatomic ligands, thereby providing concomitant insights into the structure and redox state (changes) of the [NiFe] center [14–17].

In this work, we have combined SEIRA spectroscopy, AFM, and PFV with MD simulations to elucidate enzyme-surface interactions, thereby providing comprehensive insights into a bioelectronic hybrid system consisting of the oxygen-tolerant *Re* MBH immobilized on Au electrodes coated with a SAM consisting of 6-amino-1-hexanethiol. The present integral approach is shown to successfully identify those parameters that control enzyme adsorption and orientation on electrodes, thereby ensuring structural integrity and highest catalytic efficiency.

## Materials and Methods

### 2.1 Sample Preparation

*Re* was cultured and MBH was purified as described in Goris *et al* [6]. SAM-formation was achieved by incubation of the Au electrode with 1 mM amino-1-hexanethiol in ethanol for 12 hours. The *Re* MBH was adsorbed by incubating the SAM-covered Au electrode with 1 μM protein solution in 10 mM potassium phosphate buffer at pH 7 for 30 min at 4°C.

### 2.2 SEIRA measurements

SEIRA measurements were performed in the Kretschmann-ATR configuration using a Si prism with an angle of incidence of 60°. Thin, nano-structured Au films were formed on the flat surface of the Si prism by electroless (chemical) deposition. SEIRA spectra were recorded from 4000 to 1000 cm^−1^ with a spectral resolution of 4 cm^−1^ on a Bruker IFS 66 v/S spectrometer equipped with a photovoltaic MCT detector. 400 scans were co-added for one spectrum [14].

### 2.3 PFV measurements

PFV was performed using a μAutolab potentiostat from Metrohm equipped with a three electrode set-up consisting of a Ag|AgCl reference electrode (3.0 M KCl), a Pt mesh as counter electrode and an Au working electrode. All potentials are reported with respect to the standard hydrogen electrode (SHE) [ca. 210 mV higher compared to those determined experimentally using an Ag|AgCl (3.0 M KCl) reference electrode]. Prior to SAM formation, the Au surface was cleaned electrochemically in 0.1 M H_2_SO_4_ solution by cycling six times between +210 mV and +1610 mV at a scan rate of 50 mV s^−1^. Current densities *j* (μA cm^−2^) are reported relative to the real surface area of the electrode, determined from the Au-oxide surface reduction method described elsewhere [18]. All experiments were performed under Ar or H_2_ gas atmospheres (high-purity grade 5.0, with implemented oxygen filters from Agilent) in the SEIRA setup described previously [14]. 10 mM potassium phosphate buffer at pH 5.5 was used as electrolyte solution. The temperature for PFV was set to 25°C by using a thermostat from Thermo Fisher Scientific. All experiments were reproduced at least three times.

### 2.4 AFM measurements

The AFM images were recorded on a commercial instrument, Cypher AFM (Asylum Research, Santa Barbara, USA). The topographic mapping was performed in non-contact-mode with a standard tip (7 nm in diameter) purchased from Olympus. Considering the diameter of the AFM tip, information on the size of the surface adsorbed enzymes is gained by the lateral periodicity rather than the height of the scanning z profile. Au coated Si-wafers for AFM measurements were purchased from Sigma Aldrich. The thickness of the Au layer was 100 nm, Ti served as adhesion layer between silicon and gold.

### 2.5 MD Simulations

The initial structural model was constructed on the basis of the *Re* MBH crystal structure (reduced HoxGK heterodimer, pdb entry: 3RGW), taking into account the correct configuration of the active site diatomic ligands [7,8] and the C-terminal hydrophobic tail of the MBH small subunit carrying the *Strep*-tag II peptide. The C-terminal extension, not resolved in the crystal structure, was modelled as an α-helical chain (see S1 Appendix, section 2.1 for further details). The SAM-coated Au surface was built as described previously [19], assuming a partial protonation (8%) of the amino groups, in line with the approximate p*K*_a_ (6.0±0.2) of 6-amino-1-hexanethiol in Au-attached SAMs [19].

In order to roughly estimate the energetically favorable orientations of the MBH on the SAM, *in vacuo* interaction energies neglecting polarization effects were calculated using the NAMD energy plugin implemented in VMD 1.9.1. [20] The different orientations of *Re* MBH with respect to the SAM were defined by two angles θ and Ψ describing the rotation of the enzyme around the x- and y-axis, respectively. Orientation-sampling was performed in 10° steps from 0 to 360°. In all steps the protein was placed 5 Å away from the SAM. The energetically favorable states were solvated with explicit TIP3P water molecules and used as input for subsequent classical all-atom molecular dynamics (MD) simulations, a well-established technique to study protein immobilization [21]. The 50 ns long MD simulations of the solvated protein-surface systems were carried out with NAMD 2.7 [22] using the CHARMM 27 force field [23]. Further details of the simulations are given in the S1 Appendix (section 2).

## Results and Discussion

### 3.1 Adsorption process of *Re* MBH

A SAM-coated Au film deposited on a silicon prism served both as working electrode and IR signal amplifier in such way that PFV and SEIRA spectroscopy could be carried out simultaneously with the same device [14,24]. *Re* MBH carrying a *Strep*-tag II at the very end of the C-terminus of the small subunit [25] (see S1 Appendix, section 2.1) spontaneously adsorbed to the SAM-coated Au electrode from the bulk solution as proven by the increase of the spectroscopically monitored amide I and II bands (Fig 1A). This result is in good agreement with previous spectroscopic studies on polyhistidine-tagged *Re* MBH [14]. In addition, the corresponding CO and CN stretching modes of the active site were probed at frequencies characteristic of the Ni_r_-B species. This so called “ready” oxidized state is typical for as-isolated enzyme and easily activated, yielding catalytically active *Re* MBH (Fig 1B). AFM measurements of the electrode surface after enzyme immobilization (Fig 1C and 1D) revealed a lateral periodicity of ca. 9 nm in the corresponding height-profile (see Materials and Methods part). This indicates a homogeneous surface coverage of well-distributed single *Re* MBH molecules adsorbed on the SAM-coated gold islands (surface structure, see Fig A in S1 Appendix). Consistently, ellipsometric measurements revealed an enzyme layer thickness of ca. 7 nm, indicating a protein monolayer (Fig B in S1 Appendix). Based on these results, the *Re* MBH surface coverage was estimated to be 0.2 pmol cm^−2^ (see S1 Appendix, section 1.1).

### 3.2 Enzymatic activity and stability

**Fig 2:**
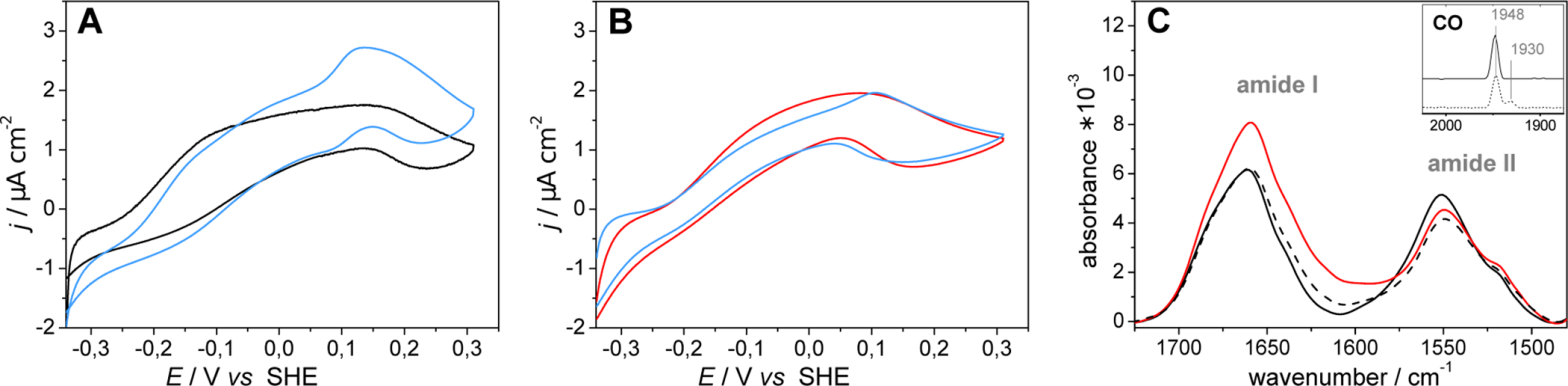
Voltammetric traces of *Re* MBH immobilized on SAM-coated Au electrodes in the presence of H_2_-saturated buffer : (A) measured before (black) and after (blue) addition of methylene blue; (B) measured before (red) and after (blue) addition of methylene blue, but subsequent to a 5 min pre-activation step at −340 mV in the presence of H_2_. All voltammetric measurements were carried out at room temperature with a scan rate of 5 mV s^−1^. (C) SEIRA spectra of the amide region recorded before (solid black), after (dashed black), and during (red) the pre-activation at −340mV under H_2_ atmosphere. The inset shows the corresponding stretching vibration of the CO ligand at the active site prior (solid line) and after (dashed line) pre-activation in the oxidized state. Black spectra (*vide supra*) were recorded at OCP (+260 mV). Potentials are given with respect to the standard hydrogen electrode (SHE).

Voltammetric traces recorded in the presence of H_2_-saturated buffer revealed current signals similar to those previously observed for *Re* MBH immobilized on graphite and Ag electrodes under H_2_ atmosphere (Fig 2A) [26,27]. The characteristic sigmoidal shape reflects the electrocatalytic activity of the immobilized enzyme for H_2_ oxidation, including the typical current drop at higher potentials, related to reversible oxidative inactivation of *Re* MBH [1,2]. These data indicate that the catalytic activity of the *Re* MBH is preserved upon adsorption on the SAM-coated Au electrode. For comparison, a cyclic voltammogram of the electrode prior to protein immobilization is shown in Fig D in S1 Appendix.

Interestingly, the orientation of the individual enzyme molecules on the electrode surface is not homogeneous, and addition of a redox mediator (methylene blue) to the buffer solution causes an increase of the catalytic current (Fig 2A, blue trace) [12]. This finding suggests that a fraction of the immobilized enzyme molecules adopts an orientation that impedes direct electron exchange with the electrode. However, when the electrode potential was set to −340 mV (*versus* standard hydrogen electrode, SHE) prior to the cyclic voltammetry studies, a slight but reproducible increase (15%) of the catalytic current related to direct electron transfer was observed (Fig 2B). This current increase was accompanied by a change of the amide I / amide II intensity ratio in the SEIRA spectrum (Fig 2C), reflecting a potential-induced re-orientation of the immobilized protein at −340 mV, which is still sufficiently positive to prevent reductive SAM desorption (see Fig C in S1 Appendix). As a result of this pre-activation step, some initially electro-inactive enzyme molecules have obviously adopted an orientation that allows direct electron transfer. Consistently, the catalytic current remained virtually unchanged upon subsequent addition of the redox mediator (Fig 2B, blue trace), pointing towards an increased amount of homogeneously oriented, electroactive adsorbed enzyme molecules. Subsequent to cyclic voltammetry measurements, the sample was treated with air-saturated buffer yielding an open circuit potential (OCP) of ca. +260 mV. Under these conditions, the re-orientation of the protein was reversed as indicated by the SEIRA spectrum (Fig 2C, dashed black line), which was similar to that measured before pre-activation under the same conditions (Fig 2C, solid black line). During the entire processes, the integrity of the active site was almost fully preserved (up to 80%), as indicated by the intensity of the absorption band corresponding to the CO stretching mode of the Ni_r_-B state (inset of Fig 2C). Only minor amounts were transformed to an irreversibly inactivated state (Ni_ia_-S) characterized by a CO stretching band at 1930 cm^−1^ [5,6].

### 3.3 Possible enzyme orientations as revealed by MD studies

To rationalize the experimental findings, classical all-atom MD simulations [28] were carried out following the procedure described above and in S1 Appendix. Notably, the *Re* MBH is characterized by a weaker dipole moment than O_2_-sensitive ‘standard’ hydrogenases (680 *versus* 1050 Debye, Fig E in S1 Appendix),[19] and it lacks a distinct negatively charged surface patch. These two aspects preclude an intuitive prediction of a preferred electrostatically determined binding orientation. Thus, a protein-surface interaction energy landscape was constructed (also see S1 Appendix, section 2.3 and Fig F in S1 Appendix), and the two energetically most favorable orientations of *Re* MBH on the SAM-coated electrode served as initial states for the MD simulations (Fig G in S1 Appendix).

**Fig 3:**
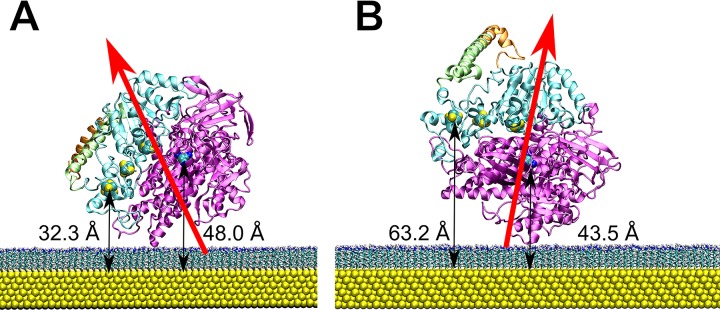
Two final orientations (A, B) of the *Re* MBH adsorbed on the SAM-coated Au surface, as predicted by the MD simulations. The protein backbone of the large and small subunit is colored in magenta and cyan, respectively. Atoms of the FeS clusters (yellow/white) and the [NiFe] active site (dark blue/cyan) are indicated as spheres. The C-terminus of the small subunit and the *Strep*-tag II are highlighted in green and orange, respectively. The SAM is depicted as blue-tipped sticks and the Au film as yellow spheres. The resulting overall dipole moments of the differently adsorbed enzyme molecules are displayed as red arrows.

These subsequent, 50-ns long MD simulations describing the initial adsorption revealed the existence of at least two stable configurations of *Re* MBH on the surface, termed A and B in the following (Fig 3). While further re-orientations on larger time scales cannot be entirely excluded, the stability of these states is indicated by the *quasi* static orientation of the enzyme’s dipole moment with respect to the surface (Fig 3 and Fig H in S1 Appendix). In both cases, electrostatic repulsion led to a movement of the entire C-terminal tail away from the surface (Fig 3). In addition, the presence of the surface significantly reduces the directional fluctuation of the enzyme’s dipole moment (Fig H in S1 Appendix), while its overall strength increases during the adsorption process (Fig I in S1 Appendix). This finding highlights the necessity to consider the entire enzyme-surface system in the MD simulation.

Furthermore, the backbone root-mean square deviation (RMSD) values of the large and small subunits of *Re* MBH were found to be less than 3.5 Å compared to the crystal structure (Fig J in S1 Appendix). In line with the SEIRA spectroscopic results, these low values demonstrate the structural integrity of the *Re* MBH dimer, ruling out denaturation of the immobilized enzyme for both orientations.

Analyzing these two predicted orientations, configuration A represents an orientation that is likely appropriate for a slow direct electron transfer between the distal FeS cluster of the *Re* MBH and the electrode, which are separated by 32.3 ± 1.4 Å (Fig 3A). Interestingly, this distance is much larger for orientation B as the exit of the electron transfer chain points away from the surface (Fig 3B). Albeit closer than in orientation A, also the active site of configuration B resides outside electron tunneling distance to the electrode (43.5 ± 0.3 Å in B *versus* 48.0 Å ± 0.7 Å in A, taking the Ni atom as reference). Clearly, the electrocatalytic processes of *Re* MBH in orientation B would require a mediator in the bulk solution to shuttle electrons between the distal FeS cluster and the electrode. Consequently, orientations A and B might be attributed to the fractions of experimentally observed *Re* MBH molecules (at OCP, ca. +260 mV) undergoing direct and mediated electron exchange with the electrode, respectively. Notably, relative amounts of the two orientations are controlled by local electrostatic interactions with the SAM-coated electrode and the tendency to align the molecular dipole moment in the interfacial electric field. The latter contribution changes significantly below the potential-of-zero-charge (PZC) of the SAM-coated Au electrode, which is ca. −250 mV (Fig C in S1 Appendix). This explains the experimentally observed pre-activation at −340 mV, where mediated electron transfer becomes negligible. Thus, we conclude that an A-like orientation prevails under these conditions. This potential-dependent re-orientation of the adsorbed *Re* MBH is also consistent with the observed changes of the amide I / amide II intensity ratio in the SEIRA spectra measured at OCP and at the pre-activation potential (Fig 2C).

## Conclusions

In summary, we applied an integral approach of SEIRA spectroscopy, AFM, and PFV in combination with MD simulations to study immobilization, structural integrity, and catalytic activity of an oxygen-tolerant [NiFe] hydrogenase on SAM-coated Au electrodes. The experimental data indicate different orientations of the *Re* MBH biomolecules initially adsorbed on the electrode surface. We identified at least two enzyme populations that operate with different types of electronic communication with the electrode, i.e. direct and mediated electron transfer. The initial heterogeneous distribution can be rationalized on the basis of MD simulations which, despite the relatively short simulation times, seem to provide a good model for the immobilized enzyme. Through pre-activation at negative potentials, below the PZC of the SAM-coated Au electrode, the fraction of the electro-active molecules exhibiting direct electron transfer was increased due to protein re-orientation, eventually resulting in a higher catalytic current. In the light of these findings, the presented experimental and theoretical approach promises to be of general applicability to study and optimize bioelectronic devices with different enzymes, modes of protein adsorption, and support materials.[29]

## Supporting Information

S1 Appendix Complementary experimental AFM, elipsometric, and electrochemical data as well as computational details. (PDF)
