# Supplementary material for: Orientation-Controlled Electrocatalytic Efficiency of an Adsorbed Oxygen-Tolerant Hydrogenase
Source: PLoS One. 2015 Nov 18;10(11):e0143101. doi: 10.1371/journal.pone.0143101 (PMC4651547; doi:10.1371/journal.pone.0143101)
Supplement: S1 Appendix — (PDF) [file pone.0143101.s001.pdf]

S1 Appendix

Orientation-controlled electro-catalytic efficiency of an adsorbed oxygen-tolerant hydrogenase

Nina Heidary[1¶], Tillmann Utesch[1¶], Maximilian Zerball[1], Marius Horch[1], Diego Millo[2], Johannes Fritsch[3], Oliver Lenz[1], Regine von Klitzing[1], Peter Hildebrandt[1], Anna Fischer[1,4]*, Maria Andrea Mroginski[1]*, and Ingo Zebger[1]*

[1] Institut für Chemie, Technische Universität Berlin, Straße des 17. Juni 135, D-10623 Berlin, Germany, E-Mail: *andrea.mroginski@tu-berlin.de*; *ingo.zebger@tu-berlin.de*

[2] Biomolecular Spectroscopy/LaserLaB Amsterdam, Vrije Universiteit Amsterdam, De Boelelaan 1083, NL-1081 HV Amsterdam, The Netherlands

[3] Institut für Biologie / Mikrobiologie, Humboldt Universität zu Berlin, Chausseestr. 117, D-10115 Berlin, Germany

[4] Institut für Anorganische und Analytische Chemie, Albert-Ludwigs-Universität Freiburg, Albertstrasse 21,
D-79104 Freiburg, Germany, E-Mail: *anna.fischer@ac.uni-freiburg.de*

Content

1. Complementing experimental data
   1.1. Atomic Force Microscopy – reference data of the 6-amino-1-hexanethiol Self-Assembled Monolayer (SAM)
   Figure A
   1.2. Ellipsommetry – determination of the protein layer thickness
   Figure B
   1.3. Electrochemical desorption of the bare 6-amino-1-hexanethiol SAM
   Figure C
   1.4. Protein Film Voltammetry – compared to the bare 6-amino-1-hexanethiol SAM
   Figure D

2. Simulation setup
   2.1. Hydrogenase
   2.2. Surface
   2.3. Surface – enzyme system
   2.4. Molecular dynamics protocol

3. Figures Simulation
   Figure E Structure of the membrane-bound hydrogenase (MBH) dimer
   Figure F Interaction energy landscape between the MBH and the surface
   Figure G Initial alignment of the MBH on the surface
   Figure H Evolution of the angle between the dipole moment and the surface normal
   Figure I Evolution of the dipole moment
   Figure J Evolution of the root-mean-square deviation

4. References

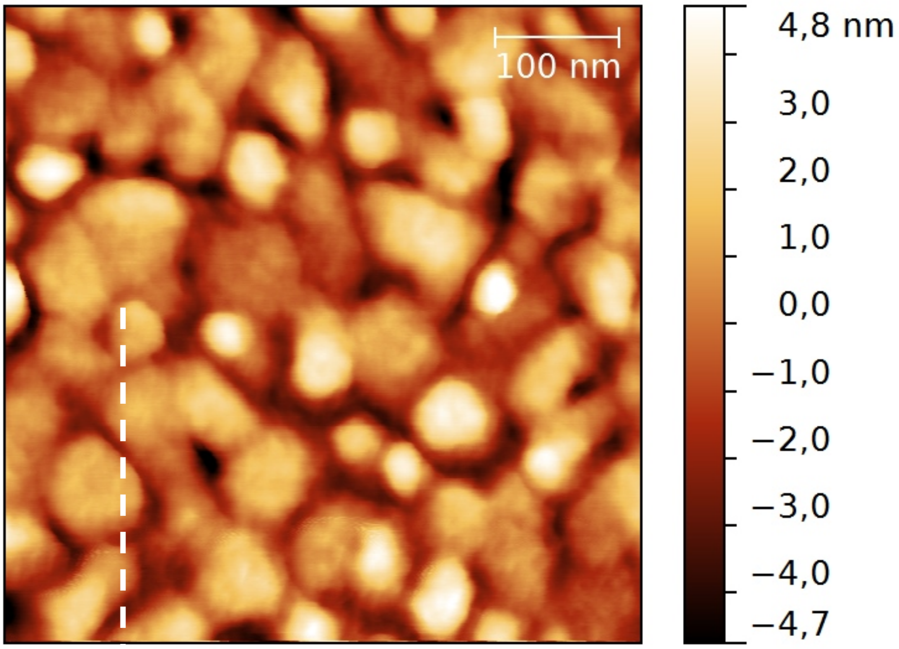

Figure A. Non-contact-mode AFM topographic mapping of the 6-amino-1-hexanethiol SAM-modified gold surface without immobilized protein. The corresponding height profile of the area, as indicated by the dashed vertical line, is shown in the manuscript in Figure 1D.

The enzyme coverage was estimated by evaluating AFM images of several independently prepared samples and at least three different spots on each sample. The amount of *Re* MBH units was counted *via* the respective periodicity derived from the height profiles as indicated in Figure 1D for all recorded AFM images and was averaged subsequently.

1.2. Ellipsometry – determination of the protein layer thickness

Ellipsometry has been applied to determine the thickness of the protein layer adsorbed on gold electrodes coated with an amino-terminated Self-Assembled Monolayer (SAM). The respective ellipsometric measurements were performed with a PCSA (polariser-compensator-sample-analyzer) ellipsometer (Optrel GbR, Sinzing, Germany). The experiments were carried out at a constant wavelength of 632.8 nm and a fixed angle of incidence of 70° (near the Brewster angle of the Si/air interface). The layer thickness was calculated with the software "Ellipsometry: simulation and data evaluation" (Optrel, v. 3.1). For the protein, a one-box model was assumed, in which the continuum media were air (n = 1.000) and gold (n = 0,144; k = 3,178). The respective refractive index n and the absorption constant k of gold were determined by measuring 5 different spots on the bare gold surface. With respect to its small thickness the SAM layer could be neglected. In such way the thickness of the protein layer was fitted. The obtained average ellipsometric angles Δ and Ψ were determined to be 100.19° and 43.29°. As thin films (< 10 nm) were investigated, only the respective Δ values change significantly. The refractive index of the enzyme layer n was estimated to a value of 1.48.[1] Figure A displays changes of the ellipsometric angle Δ in dependence of the thickness with respect to the applied model.

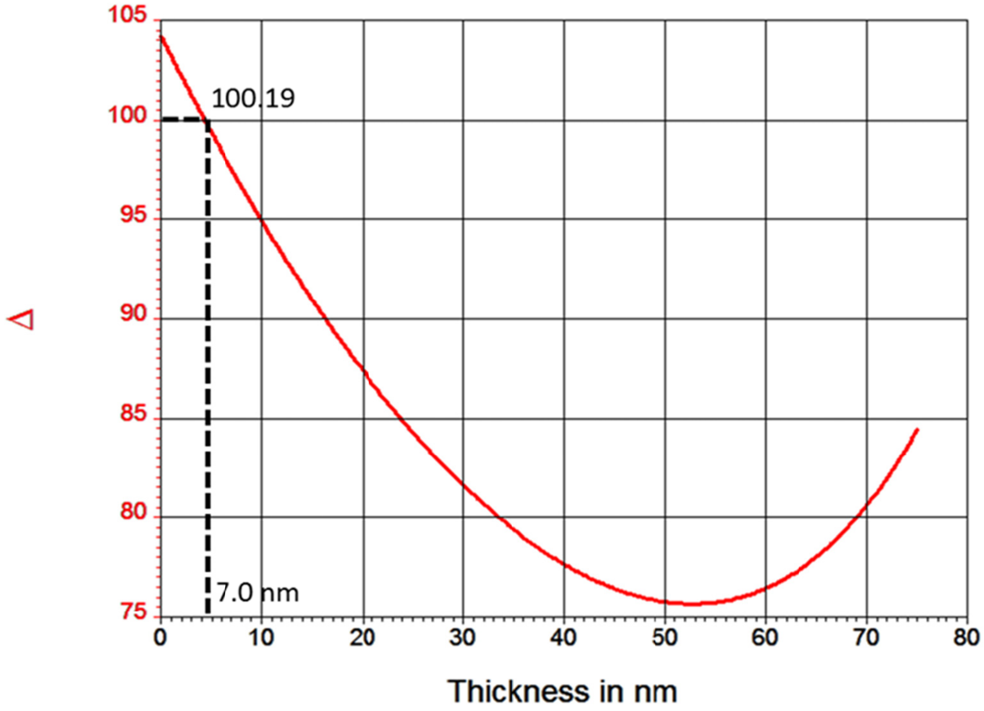

Figure B. Simulation of the ellipsometric angle Δ in dependence of the thickness according to the applied model. The measured average Δ value of 100.19° represents a thickness of ca. 7 nm.

1.3. Electrochemical desorption of the amino-1-hexanethiol SAM

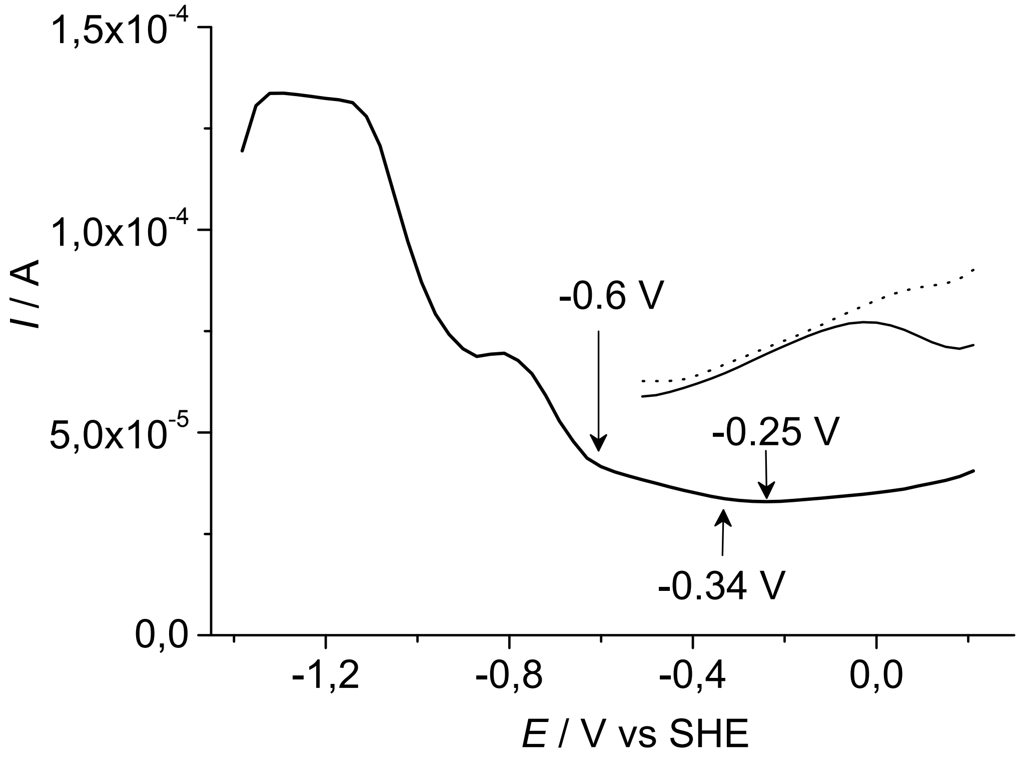

Figure C. Reductive desorption of the amino-1-hexanethiol SAM. The chemically modified Au electrode was subjected to an AC voltammetric sweep from +0.2 to −1.4 V in a buffered solution at pH 5.5 under Ar atmosphere. Scan rate, frequency, and potential amplitude were 50 mV s^{-1}, 100 Hz, and 4 mV, respectively. In the potential region between 0 and −0.8 V, the amplitude of the capacitive current of the electrical double layer (*I*) is essentially constant, and its value is about half that of the bare Au electrode, hereby used as a blank (dotted line). At poised potentials lower than −0.6 V, the current increases exponentially, as expected for SAM desorption.[2] The capacitive current measured after SAM desorption (short thick line) is twice that of the Au-SAM electrode and similar to that of the blank (dotted line). Please note that the minimum of the AC trace at −250 mV for the SAM-coated electrodes corresponding to the point of zero charge of the system is very close to −340 mV.

1.4. Protein Film Voltammetry – compared to the bare 6-amino-1-hexanethiol SAM

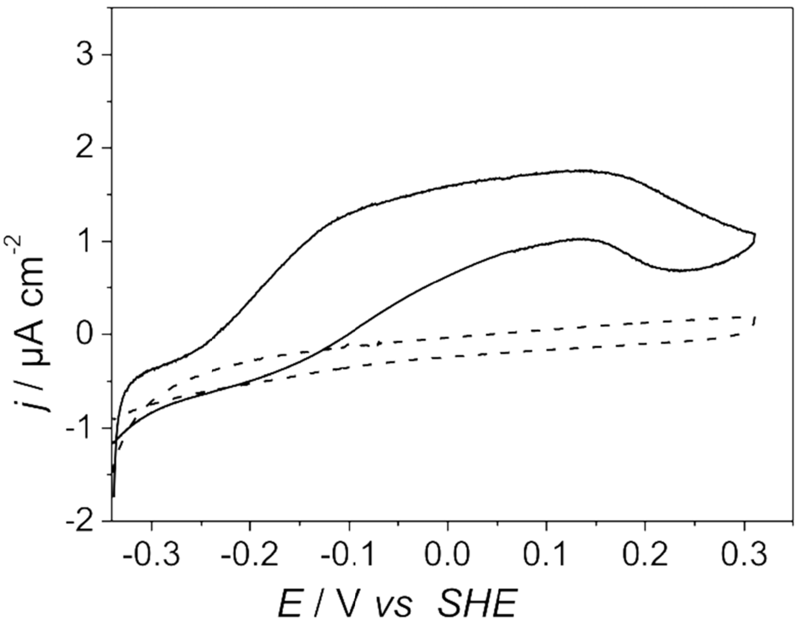

Figure D. Voltammetric traces of immobilized *Re* MBH on a SAM-coated Au electrode in the presence of H_2-saturated buffer (solid line), corresponding to Figure 2A, and the respective amino-1-hexanethiol SAM-coated Au electrode prior to immobilization (dashed line).

SI 2: Simulation setup

2.1 Hydrogenase

The structure of reduced MBH from *Ralstonia eutropha* (PDB: 3RGW) (figure E) served as a starting point for the calculations. The structure consists of the small subunit harbouring three FeS clusters and the large subunit carrying the active site and a conserved Mg^{2+} ion. The correct ligand configuration of the active site, as determined recently,[2] was taken into account. Furthermore, all crystallographic water molecules were included in the MBH model. The protein matrix was protonated according to pH 7.0 and treated with the CHARMM 27 force field.[3] All histidine residues were modelled as HSD carrying a proton at the δ-nitrogen of the imidazole ring. The FeS clusters and the active site were treated as rigid bodies by constraining their internal motions. Their non-bonding interactions were calculated as reported earlier,[2] where the partial charges were derived by electrostatic potential fits with the Merz-Singh-Kollmann scheme.[4],[5]

Moreover, the small subunit of the MBH hetero dimer, HoxK, was elongated by its C-terminal membrane anchor and an associated *Strep*-tag II peptide used for purification. The construction of the fusion protein is described in Schubert et al. [6] These structural elements are not resolved in the crystallographic structure, but the prediction with the PSIPRED server [7] showed the membrane anchor as a α-helical element, which is in agreement with the recently resolved crystallographic structure of hydrogenase 1 from *E. coli*.[8] Thus, the C-terminus was manually generated as an α-helix with VMD 1.8.7.[9] The *Strep*-tag II peptide was predicted as random coil by PSIPRED [7] and built as an extension to the C-terminal helix as predicted.

The final model was energy-minimized, heated to 300 K, and equilibrated for 10 ns with NAMD 2.7 [10] to relax the structure. During these steps performed *in vacuo*, the experimentally resolved parts were fixed to their positions and only the extension was allowed to move freely.

2.2 Surface

The gold electrode was modelled as a perfect three layer Au(111) film with the x- and y-dimensions of ca. 120 × 120 $Å^2$. The fixed Au(111) film was functionalized with 672 6-amino-1-hexanethiol SAM molecules, ca. 8 % of which were protonated and positively charged, which is in line with the approximate pK_a (6.0±0.2).[11] In order to neutralize the resulting net charge of the SAM, the bottom layer gold atoms were slightly negatively charged (−0.02777 e). All other gold atoms were treated as uncharged. In this way, a neutral surface was generated. The sulfur atoms of the SAM were arranged in a $(\sqrt{3} \times \sqrt{3})R30°$ lattice with a nearest neighbor spacing of 4.98 Å on the perfect Au(111) surface.[12],[13] The SAM molecules were initially tilted by ca. 30°. Gold and SAM atoms were described with the vdW radii from Bizzarri *et al.*[14] and the CHARMM force field for lipids,[15] respectively. The functional groups of the SAM were adopted from protonated and deprotonated lysine. Gold and SAM sulfur atoms were fixed to their positions, so that no binding parameters were required.

2.3 Surface–MBH system

In order to avoid long re-orientation times of *Re* MBH on the surface, we searched for energetically favorable starting orientations for the MD simulations. This was done in a systematic scanning of the interaction energy between *Re* MBH and the surface. The interaction energy of the enzyme (in a minimal separation distance of 5 Å with respect to the

SAM) and the surface was evaluated with the NAMD energy plugin in VMD using the parameter set described above. In each step of the scanning, the *Re* MBH was rotated by the angles Φ and Ψ around the x- and y- axis, respectively (figure F). The resulting interaction energy landscape, shown in figure F, identified two energy minima of *Re* MBH on the surface. Thus, two models starting from these energetically favorable orientations, shown in figure G, were simulated. In both scenarios, the MBH was placed in a 5 Å separation distance on the surface. Then, the two models were solvated in 120 × 120 × 150 $Å^3$ (configuration A) and 120 × 120 × 170 $Å^3$ (configuration B) large TIP3P water[16] boxes. Both systems were neutralized and 15 mM Na^+Cl^- was added.

2.4 Molecular dynamics simulations protocol

All simulations were performed with NAMD 2.7 [10] using the CHARMM 27 force field [3] as described above. First, both models were energy-minimized with the conjugated gradient algorithm and heated to 300 K. Then the water was equilibrated for 60 ps. During these steps position restraints of 25 kcal mol^{-1} $Å^{-2}$ on all heavy atoms were stepwise decreased until all atoms were allowed to move freely, except those described above. After the preparation, the two models were subjected to 50 ns long production dynamics carried out in an NPaT ensemble (constant number of particles (N), pressure (p = 1 atm), surface area (A), temperature (T = 300 K)) realized by Langevin piston dynamics.[17] Short-ranged electrostatics and vdW interactions in the periodic systems were cut at a distance of 12 Å. Long-ranged electrostatics were calculated by the Particle-Mesh Ewald summation.[18] The time step of 2 fs was enabled by using the Rattle algorithm constraining all bonds containing hydrogen atoms.

SI 3: Figures Simulation

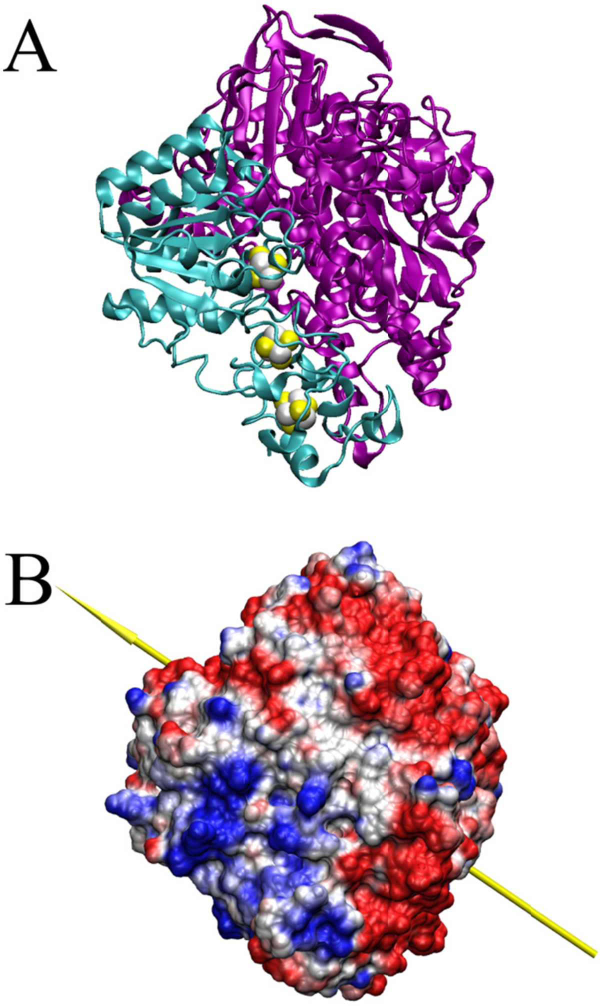

Figure E. Structure of the *Re* MBH dimer. Panel A shows the secondary structure of the enzyme. The small and large subunits are colored in cyan and purple, respectively. Additionally, the FeS clusters are indicated as spheres. In panel B, the electrostatic potential surface calculated with the APBS tool [19] is qualitatively displayed (range: –90 kT/e to +45 kT/e). Red and blue indicate negatively and positively charged regions, respectively. The yellow arrow indicates the direction of the dipole moment (ca. 680 Debye) of the dimer. Charges and radii were used as defined in the CHARMM force field.

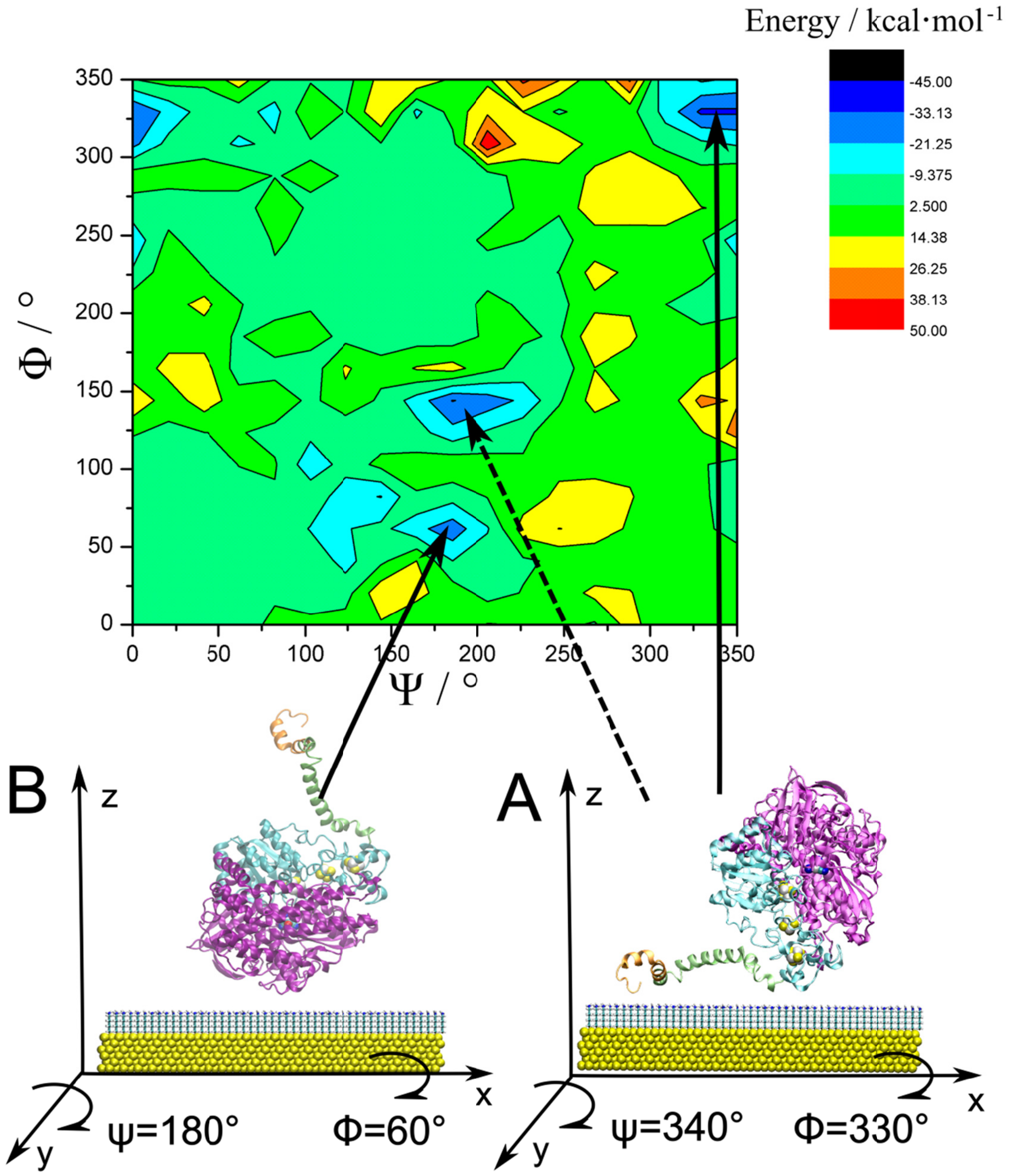

Figure F. Interaction energy landscape between the *Re* MBH and the surface. The *in vacuo* interaction energy between the rotating *Re* MBH (in a 5 Å separation distance to the SAM) and the surface was calculated with the NAMD energy plugin for VMD. In addition to the energy maps, the two energetically most favorable configurations (A and B) are shown. The dotted arrow indicates an additional energy minimum, which corresponds to a configuration very similar to configuration A. Interaction energies were computed with the parameter set described above. The two angles Φ and Ψ describe the rotation of the protein around the x- and y-axis, respectively.

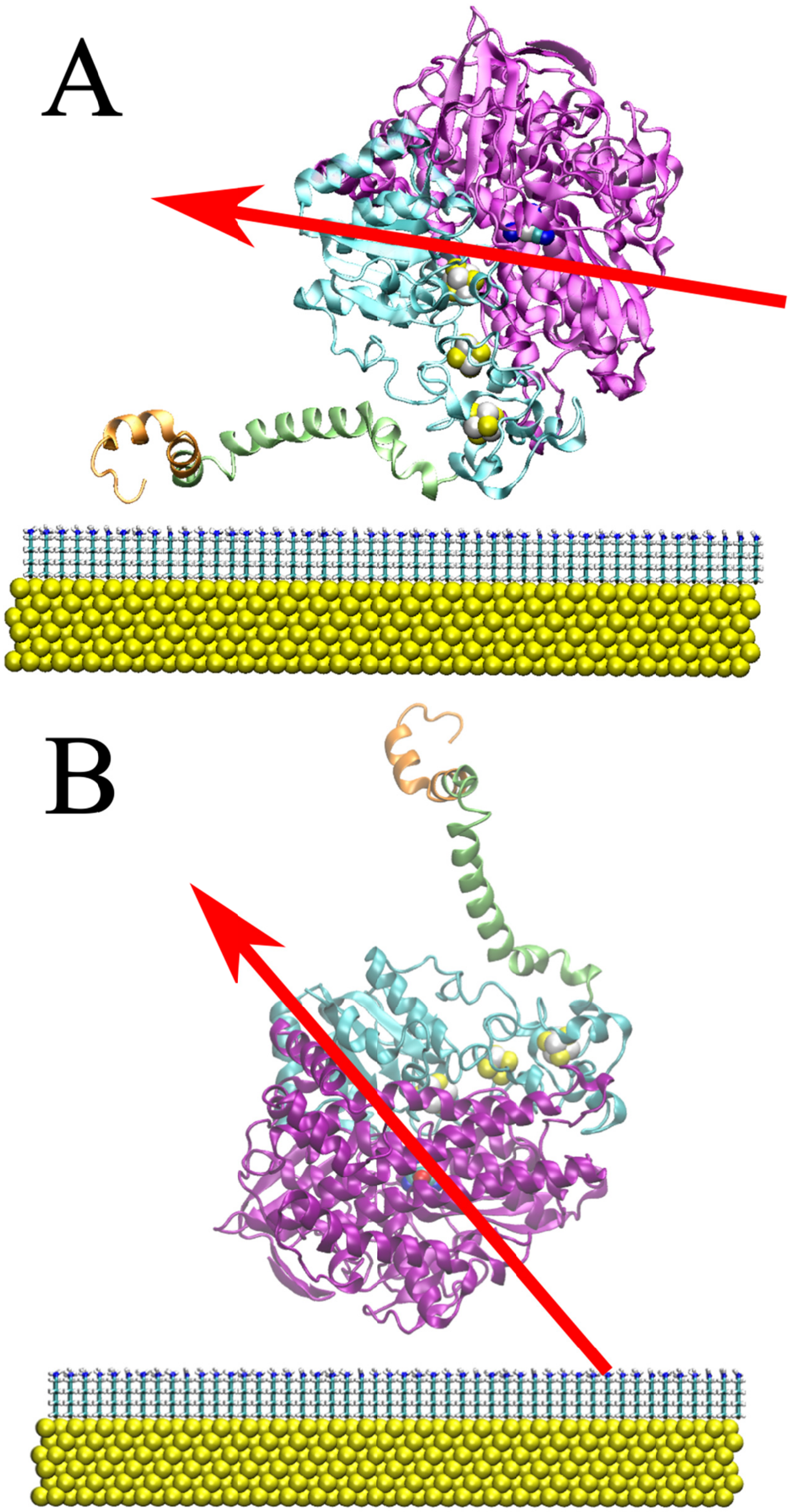

Figure G. Initial alignment of the *Re* MBH on the surface. Energetically most favorable initial orientations A and B of the *Re* MBH on the amino-terminated SAMs (Fig. F) are shown in more detail. The red arrow indicates the direction of the dipole moment of the enzyme.

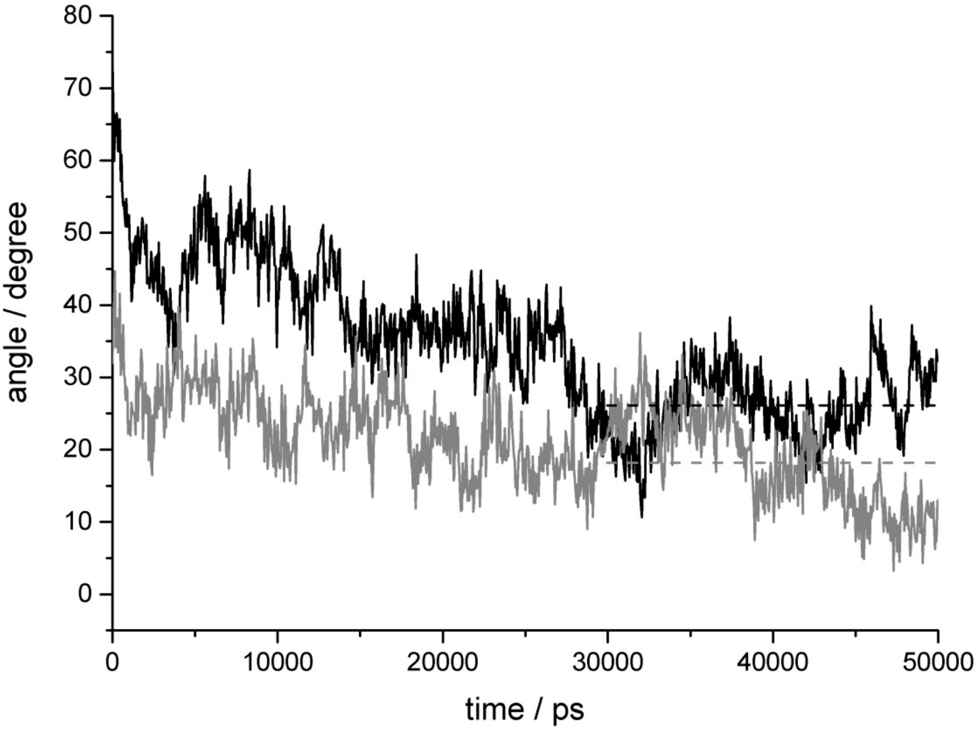

Figure H. Evolution of the angle between the dipole moment and the surface normal. The black and grey curves indicate the angle for configuration A and B, respectively. The corresponding dashed lines show the averages of the last 20 ns of the simulations.

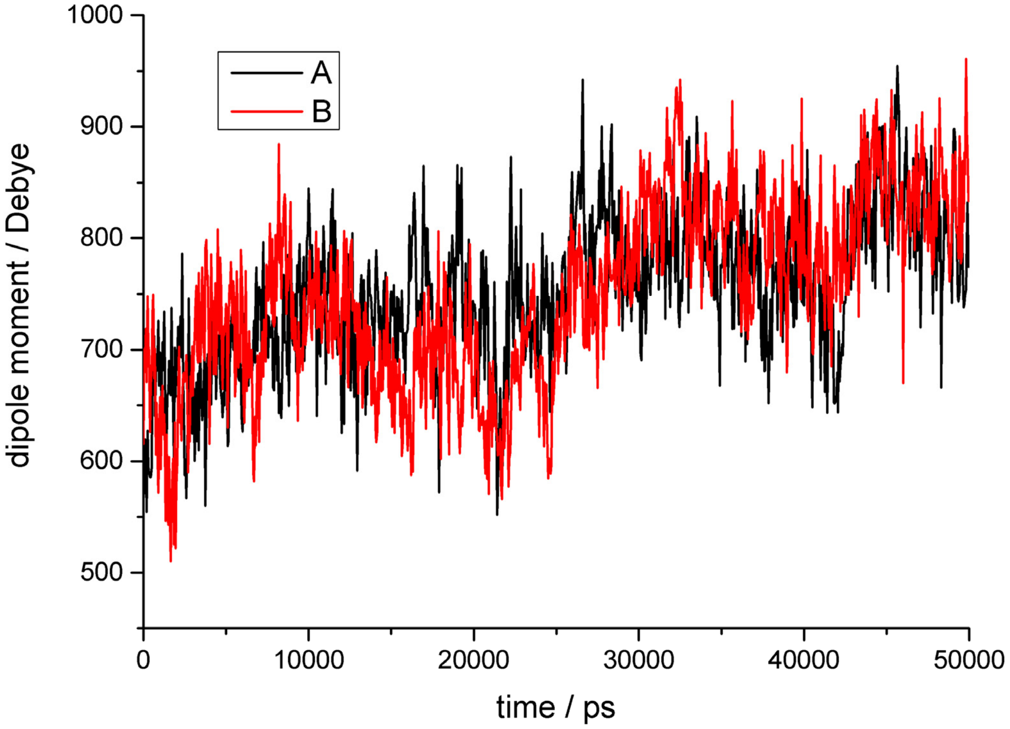

Figure I. Evolution of the dipole moment magnitude for configuration A and B.

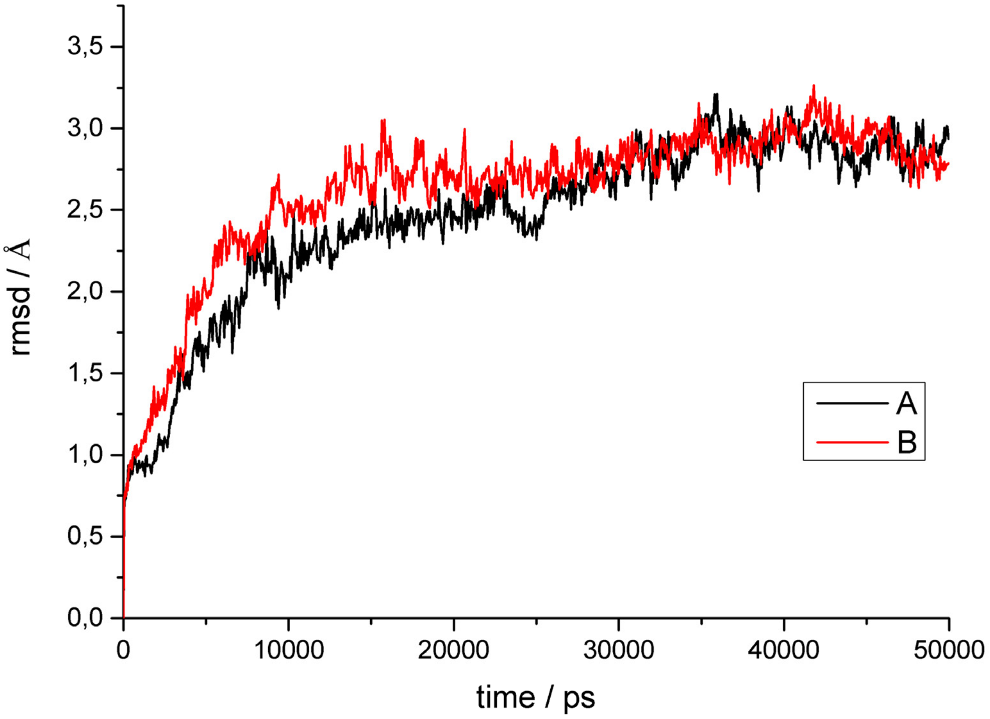

Figure J. Evolution of the root-mean-square deviation (RMSD) of the *Re* MBH backbone (without tail) for configuration A and B, calculated with respect to the crystallographic structure.

SI 4: References

1. Vörös J. The Density and Refractive Index of Adsorbing Protein Layers [Internet]. Biophysical journal. Cell Press; 2004. pp. 553–561. Available: http://linkinghub.elsevier.com/retrieve/pii/S0006349504735407

2. Rippers Y, Horch M, Hildebrandt P, Zebger I, Mroginski MA. Revealing the Absolute Configuration of the CO and CN− Ligands at the Active Site of a [NiFe] Hydrogenase. ChemPhysChem. WILEY-VCH Verlag; 2012;13: 3852–3856. doi:10.1002/cphc.201200562

3. MacKerell, AD, Bashford D, Dunbrack, RL, Evanseck JD, Field MJ, Fischer S, et al. All-Atom Empirical Potential for Molecular Modeling and Dynamics Studies of Proteins. J Phys Chem B. American Chemical Society; 1998;102: 3586–3616.

4. Singh UC, Kollman PA. An approach to computing electrostatic charges for molecules. J Comput Chem. John Wiley & Sons, Inc.; 1984;5: 129–145. doi:10.1002/jcc.540050204

5. Besler BH, Merz KM, Kollman PA. Atomic charges derived from semiempirical methods. J Comput Chem. John Wiley & Sons, Inc.; 1990;11: 431–439. doi:10.1002/jcc.540110404

6. Schubert T, Lenz O, Krause E, Volkmer R, Friedrich B. Chaperones specific for the membrane-bound [NiFe]-hydrogenase interact with the Tat signal peptide of the small subunit precursor in Ralstonia eutropha H16. Mol Microbiol. Blackwell Publishing Ltd; 2007;66: 453–467. doi:10.1111/j.1365-2958.2007.05933.x

7. Buchan DWA, Minneci F, Nugent TCO, Bryson K, Jones DT. Scalable web services for the PSIPRED Protein Analysis Workbench. Nucleic Acids Res . 2013;41 : W349–W357. doi:10.1093/nar/gkt381

8. Volbeda A, Darnault C, Parkin A, Sargent F, Armstrong FA, Fontecilla-Camps JC. Crystal Structure of the O2-Tolerant Membrane-Bound Hydrogenase 1 from Escherichia coli in Complex with Its Cognate Cytochrome b. Structure. 2013;21: 184–190. doi:http://dx.doi.org/10.1016/j.str.2012.11.010

9. Humphrey W, Dalke A, Schulten K. VMD: Visual molecular dynamics. J Mol Graph. 1996;14: 33–38.

10. Phillips JC, Braun R, Wang W, Gumbart J, Tajkhorshid E, Villa E, et al. Scalable molecular dynamics with NAMD. J Comput Chem. Wiley Subscription Services, Inc., A Wiley Company; 2005;26: 1781–1802.

11. Utesch T, Millo D, Castro MA, Hildebrandt P, Zebger I, Mroginski MA. Effect of the Protonation Degree of a Self-Assembled Monolayer on the Immobilization Dynamics of a [NiFe] Hydrogenase. Langmuir. 2013;29: 673–682. doi:10.1021/la303635q

12. Widrig CA, Alves CA, Porter MD. Scanning tunneling microscopy of ethanethiolate and n-octadecanethiolate monolayers spontaneously absorbed at gold surfaces. J Am

Chem Soc. American Chemical Society; 1991;113: 2805–2810. doi:10.1021/ja00008a001

13. Love JC, Estroff LA, Kriebel JK, Nuzzo RG, Whitesides GM. Self-Assembled Monolayers of Thiolates on Metals as a Form of Nanotechnology. Chem Rev. American Chemical Society; 2005;105: 1103–1170. doi:10.1021/cr0300789

14. Bizzarri AR, Costantini G, Cannistraro S. MD simulation of a plastocyanin mutant adsorbed onto a gold surface. Biophys Chem. 2003;106: 111–123. Available: http://www.sciencedirect.com/science/article/B6TFB-49HSV63-1/2/3ade2c410a6a10623108a2d00c3f786d

15. Feller SE, Gawrisch K, MacKerell AD. Polyunsaturated Fatty Acids in Lipid Bilayers: Intrinsic and Environmental Contributions to Their Unique Physical Properties. J Am Chem Soc. American Chemical Society; 2001;124: 318–326. doi:10.1021/ja0118340

16. Jorgensen WL, Chandrasekhar J, Madura JD, Impey RW, Klein ML. Comparison of simple potential functions for simulating liquid water. J Chem Phys. AIP; 1983;79: 926–935.

17. Feller SE, Zhang Y, Pastor RW, Brooks BR. Constant pressure molecular dynamics simulation: The Langevin piston method. J Chem Phys. AIP; 1995;103: 4613–4621.

18. Darden T, York D, Pedersen L. Particle mesh Ewald: An N log(N) method for Ewald sums in large systems. J Chem Phys. AIP; 1993;98: 10089–10092.

19. Baker NA, Sept D, Joseph S, Holst MJ, McCammon JA. Electrostatics of nanosystems: Application to microtubules and the ribosome. Proc Natl Acad Sci. 2001;98: 10037–10041.
